# Supplementary Posteromedial Plating for the Fixation of Complex Tibial Plateau Fractures: A Prospective Study

**DOI:** 10.7759/cureus.33797

**Published:** 2023-01-15

**Authors:** Manoj K Arya, Raj K Bhartiya, Santosh Kumar Singh, Pranav Sharma

**Affiliations:** 1 Orthopaedics, Shaikh-Ul-Hind Maulana Mahmood Hasan Medical College, Saharanpur, IND; 2 Orthopaedics, Maa Vindhyavasini Autonomous State Medical College, Mirzapur, IND; 3 Orthopaedics and Traumatology, Employee's State Insurance Corporation Hospital, Jajmau, Kanpur, IND; 4 Orthopaedics, Rani Durgavati Medical College, Banda, IND

**Keywords:** ptpa, posterior tibial plateau angle, ptpf, ctpf, posterior tibial plateau fractures, dual plating, posteromedial buttress plating, posteromedial plating, bicondylar tibial plateau fractures, tibial plateau fractures

## Abstract

Introduction

The management of complex tibial plateau fractures (CTPF) involving the posterior tibial plateau remains challenging and achieving maintenance of an axially stable construct with a single lateral locked plate is uncertain. Dual plating for such fractures via separate incisions can provide better fixation with superior clinical and radiological outcomes. This prospective study aimed to evaluate the results of the management of Schatzker type V and VI complex tibial plateau fractures using a conventional anterolateral plate along with a posteromedial buttress plate via two separate approaches and the potential complications associated with it.

Methods

Fifty-six patients presenting with tibial plateau fractures to the Department of Orthopaedics at a tertiary care center in the northern part of the state of Uttar Pradesh, India, between January 2018 and July 2022 were screened. Subsequently, 28 patients with CTPFs (AO/OTA types 41C1, 41C2, AND 41C3) were included in the study, managed with dual plating, and followed up for a duration of 12 months. The clinico-radiological outcome was assessed using Rasmussen's Functional Grading System (RFS), Oxford Knee Score (OKS), knee range of motion achieved, and Rasmussen's Radiological Scoring System (RRS), and statistical analysis of the data was performed.

Results

A total of 24 (85.71%) patients had excellent OKS and good to excellent RFS at the final follow-up. The average knee range of motion was 3.21° to 122°, with only two patients reporting an extensor lag of more than 10°. The final follow-up radiographs showed a mean medial proximal tibial angle (MPTA) of 83.98° ± 6.89 (75.44-89.21) and a mean posterior tibial plateau angle (PTPA) of 12.31 ± 4.69 (5.12 to 16.49) with the RRS showing excellent or good radiographic results with a mean score of 14.1 ± 1.7 (range 8-16). None of the patients showed signs of deep infection, whereas superficial infection was reported in two patients. A single case of secondary loss of particular reduction was seen.

Conclusion

Supplementary posterior buttress plating, along with the conventional anterolateral plate for the management of CTPF, achieves rigid fixation with superior articular reduction, a high knee score, a good range of motion, lower complication rates, and limited deformities with a good radiological outcome, with a few demerits of prolonged operative time, technically demanding procedure, increased blood loss, and a protracted hospital stay which can be minimized in most instances using minimally open reduction techniques and careful soft-tissue handling.

## Introduction

The operative treatment of complex tibial plateau fractures (CTPF), which involve the posterior tibial plateau, remains challenging to most orthopedic surgeons [1]. Posterior tibial plateau fractures (PTPFs), whether medial or lateral, are quite prevalent and occur in 28.8% of bicondylar tibial plateau fractures [2]. Tibial plateau fractures encompass a wide range of injuries, ranging from simple to complex fracture patterns, all of which are induced by high-energy trauma [1, 2]. The number of PTPFs has risen in tandem in recent years with the rise in traffic accidents due to high-energy injuries [3-5]. Tibial plateau fractures (which account for 30% of all tibial fractures) are classified using the Schatzker or Association for Osteosynthesis/Orthopaedics Trauma Association (AO/OTA) classifications [6, 7]. Bicondylar tibial plateau fractures, which fall under the Schatzker types V/VI or AO/OTA type 41-C, account for 18%-39% of all tibial plateau fractures and are unstable and serious injuries, with considerable articular depression, multiple displaced condylar fracture lines, meta-diaphyseal fracture extensions or comminutions, and significant deterioration of the soft-tissue envelope which may result in superficial or deep infection and skin necrosis and make them prone to compartment syndrome and vascular injuries [3, 4]. These primary injuries frequently involve the menisci and the ligaments of the knee joint and, if left untreated, lead to the development of knee instability [5]. Because of their high frequency, a large area of the joint involved, and significant displacement pattern suggesting instability of posteromedial tibial plateau fractures, surgeons should be aware of its fracture morphology and the potential need to fix this fragment using a posteromedial-based technique [4, 5]. Posterolateral tibial plateau fractures (incidence 15%) involve a mean of 14.3% of the tibial plateau [7]. Internal and external fixing methods are required to treat these fractures. Conservative management by traction and cast was very common before the advent of internal and external fixation devices. However, the prognostic outcome was poor due to complications of stiffness and malunion [3, 4]. For optimum treatment of these fractures, a general outline must be obtained by evaluating the mechanism and energy of the sustained injury, as well as a complete medical history, and thereafter, the fixation of the underlying fracture pattern should be planned by various operative options including definitive external fixation, less invasive stabilization system (LISS), dual plating with a single incision, and dual plating with a two-incision technique [8, 9]. A hybrid external fixator and staged treatment with a temporary external fixator and subsequent definitive surgery are used for fracture fixation associated with severe soft-tissue insult. External fixation devices generally are associated with pin site infection and poor patient compliance [9, 10].

As the surgical approaches and fixation systems are debatable for such fracture morphologies, there is no consensus yet on the best treatment methodology for them. Using a single lateral locking plate to fix CTPFs provides stability, avoiding soft tissue stripping and reducing operating time. However, maintaining the axial alignment is difficult when the articular component of the posterior plateau has a coronal fracture line [10, 11]. The stabilization of CTPFs associated with PTPF has improved as contemporary concepts and techniques have evolved in recent years, allowing better knowledge of precise radiological fracture geometry and fixation of the posteromedial and posterolateral fragments through two-incision dual plating using locking or buttress plate, leading to better clinical outcomes. Different studies have favored this in terms of biomechanics, easy access to the articular surface for anatomic joint reduction, adequate fixation, maintenance of alignment, and early rehabilitation to achieve a better functional outcome in addition to preventing postoperative tibial subluxation, medial collapse, and subsequent varus collapse [7-11]. Galla M and Lobenhoffer P used a direct posteromedial approach, but the PTPFs may not be assessed properly via this approach [12]. Carlson DA used posteromedial and posterolateral incisions to treat these types of fractures [13]. It is worth noticing, however, that the main limiting factor for dual plate fixation through two different incisions is extensive soft tissue dissection, leading to a higher incidence of wound complications [14, 15]. Therefore, the aim of this prospective study was to evaluate the clinical and radiological outcomes of CTPFs associated with PTPFs (AO/OTA types 41C) treated by dual plating (conventional anterolateral plate with supplementary posteromedial plate) via two separate incisions, as well as the complications that accompanied it.

## Materials and methods

Study design and objective

This prospective study was conducted to evaluate the outcomes achieved with the use of dual plating via two separate incisions in the management of CTPFs associated with PTPFs, with a short-term follow-up of a minimum of 12 months.

Study population

**Figure 1:**
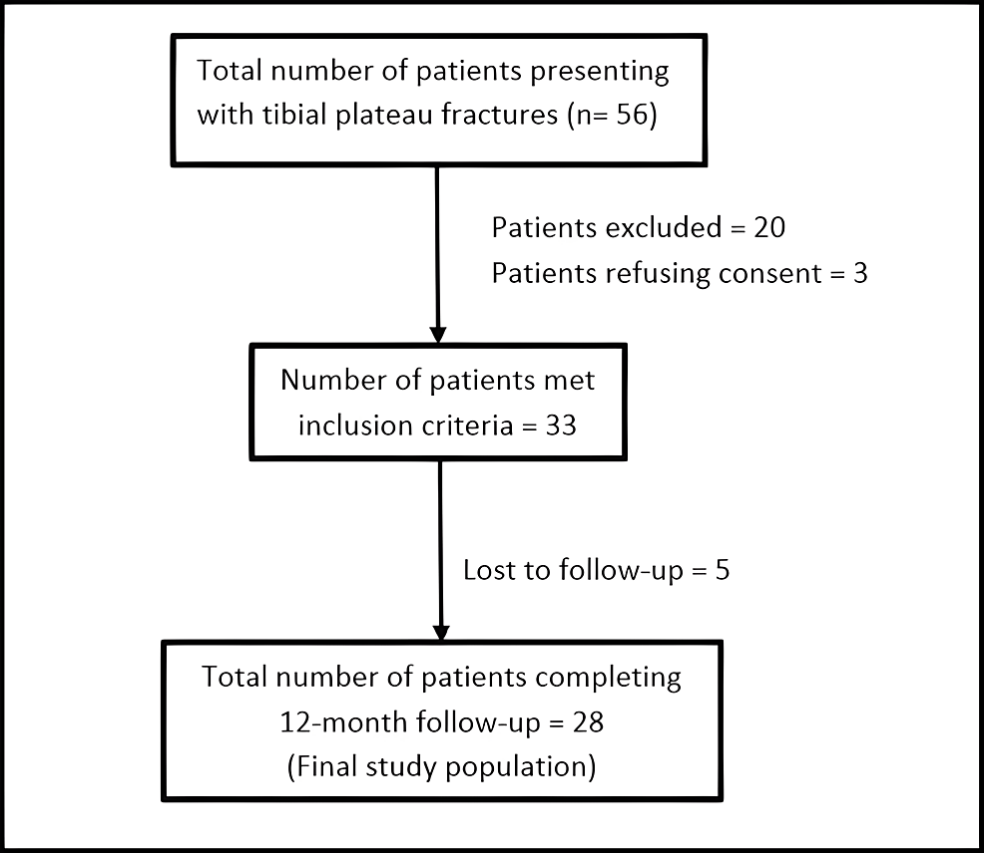
Flowchart depicting patient enrollment and the final study sample size.

Between January 2018 and July 2022, 56 patients diagnosed with tibial plateau fractures were admitted to the Department of Orthopaedics at a tertiary care center in the northern part of Uttar Pradesh, India. Of these, 33 patients diagnosed with CTPF associated with PTPF and who met the study's inclusion criteria were enrolled as study participants. All these patients were managed with dual plating (conventional lateral plate with supplementary posteromedial plate) using two different incisions. Five patients were lost during the required minimum of 12 months of follow-up and were thence excluded from the study. A total of 28 patients were included in the final study population. The flow chart showing patient enrollment in the study is depicted in Figure 1.

Ethical clearance was obtained from the Institutional Ethical Committee (IEC) prior to the start of the study (RMCS/IEC/29, Institutional Ethical Committee, SMMH Medical College Saharanpur). Informed written consent was taken from all the patients prior to their enrollment in the study.

Inclusion Criteria

1. Skeletally mature patients between 18 years and 70 years of age; 2. CTPFs associated with PTPFs, i.e., Schatzker type V, VI, or AO/OTA type 41-C.

Exclusion Criteria

1. Open fractures (Gustilo and Anderson's type II and III); 2. Pathological fractures, polytrauma patients, other chain fractures in the ipsilateral lower limb, old fractures of more than four weeks duration, and preexisting deformity; 3. Not fit for surgery in regards to anesthetic evaluation; 4. Vascular injuries and floating knee injuries; 5. Patients with follow-up of fewer than 12 months.

Preoperative evaluation

Patients presenting to the trauma emergency unit were first stabilized according to the Advanced Trauma Life Support (ATLS) protocol, and a complete clinical evaluation was done. The affected limb was examined for evidence of swelling, fractures, neurovascular status, soft tissue coverage, hemarthrosis, and limb shortening. Standard anteroposterior and lateral radiographs were obtained, and CT scans with three-dimensional reconstruction of the knee and proximal tibia were taken to determine the anatomy of the fracture. The plateau fractures were classified according to the Schatzker and the AO/OTA classification. Knee aspiration was done under aseptic conditions in patients with tensed knee joint effusion. Primary splintage of the fracture was done by either plaster of Paris slab or Bohler Braun Splint with skeletal traction (calcaneal or lower tibial). For patients with compromised and severely injured soft tissue envelopes, a treatment approach with staged procedures was adopted in which a provisional knee-spanning external fixator was applied first. Subsequently, when the soft tissue appeared receptive to surgical treatment, the fracture was definitively stabilized with plating. Limb elevation was continued after primary management, and the patient was then planned for definitive internal fixation once the tissue edema settled and a wrinkle sign appeared. All patients were operated upon by two senior orthopedic surgeons well experienced in trauma surgeries.

Operative procedure

The operation was performed first in a prone position under combined spinal with epidural anesthesia and fluoroscopic guidance, with slight knee flexion to relax the gastrocnemius muscle. A pneumatic tourniquet was used to achieve a bloodless operative field. The joint line congruity was assessed first under the guidance of an image intensifier. Definitive fixation was started on the medial side with the posteromedial approach (PM), taking a 6-8 cm long straight incision along the medial border of medial gastrocnemius starting from the medial joint line without crossing the popliteal fossa. The medial gastrocnemius muscle was retracted laterally, and the popliteus was dissected and detached subperiosteally till adequate exposure of the posteromedial fracture. To reduce the fracture hyperextension, axial traction was applied, and the fracture was reduced by pushing it with a ball spike. Fixation was performed with an angular anti-glide 3.5 mm T-buttress plate or posteromedial locking plate. Fracture stability was checked intraoperatively with the help of fluoroscopy, and wound closure was done, maintaining aseptic conditions. For the anterolateral fixation, the patient was then repositioned in a supine position, and a standard anterolateral approach (AL) with principles of minimally invasive plate osteosynthesis (MIPO) was used. Firstly, the articular reduction was assessed, and in cases with articular depression, the depressed articular surface was elevated with the help of a bone punch. Autologous bone grafting from the iliac crest was performed. If needed, a submeniscal arthrotomy through an anterolateral incision was used to achieve and confirm the anatomic reduction of the articular surface. Definitive fixation was done with a proximal tibial lateral anatomical locking compression plate. After wound lavage, closure was done in layers over a negative suction drain and compression dressing was applied.

Management of associated ligament and meniscal injury

Preoperative MRI and intraoperative examination were used to assess for the associated ligament and meniscus injuries. Ruptured menisci were repaired arthroscopically as best as possible following standard protocol. Ligamentous insertions that had been disrupted were also fixed. A lateral collateral ligament tear was suspected if the knee joint was unstable on bearing varus load on examination and was subsequently repaired. However, medial collateral ligament injuries were treated non-operatively in most instances. Anterior cruciate ligament (ACL) avulsion fractures were treated arthroscopically with suture fixation. ACL tears were managed conservatively until fracture union and assessed clinically at follow-up visits. They were managed with arthroscopic reconstruction at a later date.

Postoperative follow-up

Limb elevation and ice pack application, particularly in the first 24 hours following surgery, sought to minimize extremity edema. Postoperative standard anteroposterior and lateral X-rays of all the patients were taken. The first postoperative dressing was done 48 hours after the surgery, and the drain was removed. An angle-adjustable knee brace was used for the immobilization of the knee joint. Isometric quadriceps exercises, hamstring exercises, and passive knee range of motion were started from the second postoperative day, depending upon the stability of the fixation and the patient's tolerance to pain. An experienced physiotherapist instructed all of the patients on a regular basis. Clinical and radiological assessment was performed routinely in every follow-up visit to assess the healing process and detect any complications. At the 9th-week follow-up, patients were advised partial weight-bearing along with a range of motion and endurance exercises of the knee. Full weight-bearing was started only after the radiological union of the fracture, usually after 12 weeks. Consolidation of callus over at least three cortices was considered a union, and no signs of healing for nine consecutive months were considered nonunion. Patients were reviewed in follow-up visits at two weeks, six weeks, nine weeks, three months, six months, and one year. Patients were assessed for skin conditions, including signs of infection, deep vein thrombosis (DVT), range of movement of the knee, signs of postoperative compartment syndrome, implant failure, loss of reduction, malalignment, or any other complication related to union or soft tissue coverage.

Functional Assessment

Functional assessment was done by a designated resident doctor using Rasmussen's Functional Grading System (RFS) [16], Oxford Knee Score (OKS) [17], and knee range of motion with the help of a goniometer at the one-year final follow-up.

Radiological Assessment

**Figure 2:**
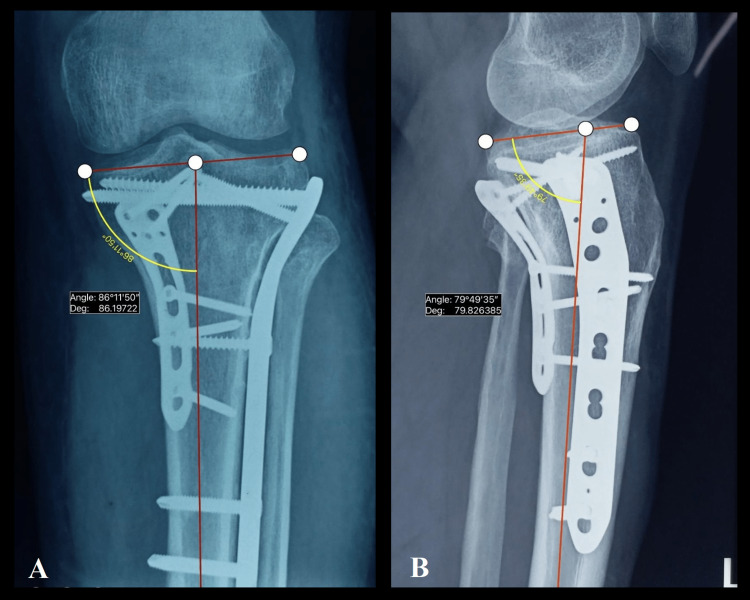
Measurement of the angles to assess the proximal tibia alignment. A: Measurement of medial proximal tibial angle (MPTA); B: Measurement of posterior tibial plateau angle (PTPA).

Rasmussen's Radiological Scoring System (RRS) was used to analyze the radiological outcomes at the one-year follow-up [18]. RRS assesses articular depression, metadiaphyseal angulation, and intercondylar widening on a scale of 6-0, with a maximum score of 6 and a minimum of 0, for a total score of 18. The posterior tibial plateau angle (PTPA) and medial proximal tibial angle (MPTA) were used to assess the proximal tibia alignment in the sagittal and coronal planes, respectively, and the PTPF reduction status. The PTPA is the angle measured between the tangential line of the tibial plateau and the perpendicular line drawn at the center of the shaft along the anterior cortex of the tibia on a true lateral radiograph of the knee. Alternatively, it can be measured by subtracting the angle between the anatomical axis of the tibia and the tangential line of the tibial plateau from 90° [19]. In AP radiographs, MPTA is the medial angle between the tangential line at the articular surface of the tibia and the anatomical axis of the tibia [20]. The measurement of MPTA and PTPA is depicted in Figure 2.

The articular surface reduction quality was assessed using lateral plain film and was classified as anatomic (0 mm step-off), acceptable (2 mm step-off), or poor (more than 2 mm step-off) [11].

From the time of surgery to the final follow-up visit, routine radiological evaluation was performed. A secondary loss of reduction was defined as a 5° increase in coronal or sagittal plane malalignment or an articular depression of 2 mm or more as compared to the initial postoperative radiograph [21].

Statistical analysis

The data were analyzed using SPSS Statistics version 22.0. (IBM Corp. Armonk, NY, USA). Basic descriptive statistics were employed to determine the frequencies and percentages of categorical variables and means and SDs for continuous variables.

## Results

The study population comprised 22 males (78.57%) and six (2.43%) female patients, with a mean age of 38.5 years (ranging from 20 to 70 years). Twenty (71.43%) patients sustained injury due to road traffic accidents, five patients (17.86%) due to falls from height, and the rest three (10.71%) patients were injured due to crushing injuries. Twenty-one patients came to the hospital within two days after injury, whereas seven reported late. Seven (25%) had Schatzker type V, and 21 (75%) had type VI bicondylar tibial plateau fracture. According to AO/OTA fracture classification, one patient had 41C1 type, 16 patients had 41C2 type, and 11 patients had 41C3 type fracture patterns. Fractures of the distal end radius, calcaneus, and lumbar vertebra were the commonly associated concomitant injuries. The average duration between injury and definitive surgery was 8.5 days (ranging from 3 to 15 days). The socio-demographic variables of the patients are depicted in Table 1.

**Table 1:** Demographic profile of the study population. RTA: Road traffic accident; AO/OTA: Arbeitsgemeinschaft für Osteosynthesefragen/Orthopaedics Trauma Association. ^1^Data expressed in mean ± SD.

Characteristics			Frequency (n)	Percentage (%)
Age (years)	20-30	38.5 ± 7.9^1^	8	28.56
	31-50		9	32.14
	41-70		11	39.29
Gender	Male		22	78.57
	Female		6	21.43
Side involved	Right		16	57.14
	Left		12	42.86
Mechanism of Injury	RTA		20	71.43
	Fall from height		5	17.86
	Crush injury		3	10.71
Schatzker fracture pattern	Type V		7	25
	Type VI		21	75
AO/OTA fracture pattern	41 C1		1	3.57
	41 C2		16	57.14
	41 C3		11	39.29
Associated fractures	Distal end radius		3	10.71
	Calcaneus		1	3.57
	Lumbar vertebra		1	3.57
	Clavicle		1	3.57
Time to surgery (days)	3-6	8.5 ± 3.9^1^	3	10.71
	7-10		19	67.86
	11-15		6	21.43

Perioperative and postoperative details of the patients are summarized in Table 2. 

**Table 2:** Perioperative and postoperative data. ^1^Data are presented as frequency (%).

Variable		Mean ± SD (Range)
Duration of surgery (minutes)		75.67 ± 5.30 (60-90)
Fixation Method^1^	Primary	25 (89.28)
	Staged	3 (10.71)
Articular Reduction^1^	Anatomic	20 (71.42)
	Acceptable	8 (28.57)
Primary Bone Grafting^1^		8 (28.57)
Submeniscal Arthrotomy^1^		4 (14.29)
Blood loss (ml)		186 ± 42.7 (130-270)
Duration of hospital stay (days)		13.7 ± 2.2 (13-35)
Time to full weight bearing (weeks)		15.3 ± 3.2 (12-20)
Time to union (weeks)		15.2 ± 1.5 (9-18)
Follow-up period (months)		22.3 ± 1.3 (15-24)

The mean duration of surgery was 75.67 ± 5.30 minutes (ranging from 60 to 90 minutes). Staged fracture fixation with the help of an external fixator first (average for seven days, range 5-10 days), followed by definitive internal dual plate fixation, was done in three patients having severe soft-tissue insult and tense swelling. The bone defects were filled with an autologous bone graft in eight patients. The anatomic articular reduction was achieved in 20 (71.42%) patients, while it was acceptable in eight (28.57%) patients. Four patients had an ACL avulsion fracture, which was addressed arthroscopically using a suture-fixation technique. One patient had an ACL body rupture, which was reconstructed arthroscopically. Five patients had medial, and two had lateral meniscus injuries, which were treated either with arthroscopic intervention or via arthrotomy incisions, depending on the location and type of injury. The lateral collateral ligament was repaired in three patients who exhibited varus instability after the fracture fixation. The average amount of blood loss was 186 ± 42.7 (130 to 270) ml. Postoperative blood transfusion was not required in any patient. The average duration of hospital stay was 13.7 ± 2.2 days (range 13-35 days). All fractures united with a mean time to union of 15.2 ± 1.5 weeks (range 9-18 weeks). The average time to allow unprotected full weight-bearing was 15.3 ± 3.2 weeks (range 12-20 weeks). Secondary procedures were not required in any patient. The mean follow-up time period was 22.3 months (range 15-24 months) with a minimum 12-month follow-up. The results of functional and radiological outcomes at the 12-month follow-up are summarized in Table 3.

**Table 3:** Functional and radiological assessment at 12-month follow-up.

S. No.	Variable	Outcome (Mean ± SD [range])
1.	Oxford Knee Score (OKS)	44.75 ± 8.5 (31-48)
2.	Rasmussen’s Functional Score (RFS)	26.95 ± 1.49 (21-29)
3.	Range of Motion	3.21° ± 2.78° - 122° ± 10°
5.	Medial Proximal Tibial Angle (MPTA)	83.98° ± 6.89 (75.44°-89.21°)
6.	Posterior Tibial Plateau Angle (PTPA)	12.31° ± 4.69 (5.12° – 16.49°)
7.	Rasmussen’s Radiological Score (RRS)	14.1 ± 2.7 ( 8-17)

The average knee range of motion was from 3.21° to 122° (range: 0°-10° for extension lag, range: 100°-135° for flexion). The mean RFS at the final follow-up was 26.95 ± 1.49. Out of 28 patients, 14 (50%) patients had an excellent result with an average score of 28, 10 (35.71%) patients had good results with an average score of 26.50, and four (14.29%) patients had fair results with an average score of 21.50. The functional outcome measured with the help of the OKS showed excellent results in 24 (85.71%) cases and good results in the rest of the four (14.29%) cases. At the final follow-up, the mean OKS was 44.75 ± 2.5 (range 37-48). The RRS showed excellent or good radiographic results with a mean score of 14.1 ± 1.7 (range 8-16). The radiographs at the final follow-up showed a mean MPTA of 83.98° ± 6.89 (75.44-89.21) and a mean PTPA of 12.31 ± 4.69 (5.12-16.49). The overall complication rate was much lower, considering fixation with two plates and two separate incisions. The various complications that occurred in this study included superficial infection in two (7.14%) cases at the lateral incision, which resolved with oral antibiotic therapy, extension lag in two (7.14%) patients, which were of <10°, and a secondary loss of reduction at six months (a 3 mm articular depression over the medial tibial plateau) in a 47-year-old patient with an AO/OTA type 41C3 fracture, although the fracture united without additional deterioration. At the final follow-up, none of the patients had any varus or valgus laxity or any anterior or posterior instability of the knee. There were no cases of deep infection or wound dehiscence, iatrogenic nerve injury, vascular complications, implant failure, malunion, or non-union over the studied time period of follow-up. The various complications have been tabulated in Table 4.

**Table 4:** Complications.

Complication	N (%)
Superficial Infection	2 (7.14)
Extension Lag (more than 10^o^)	2 (7.14)
Secondary loss of reduction (more than 2 mm articular depression)	1 (3.57)

**Figure 3:**
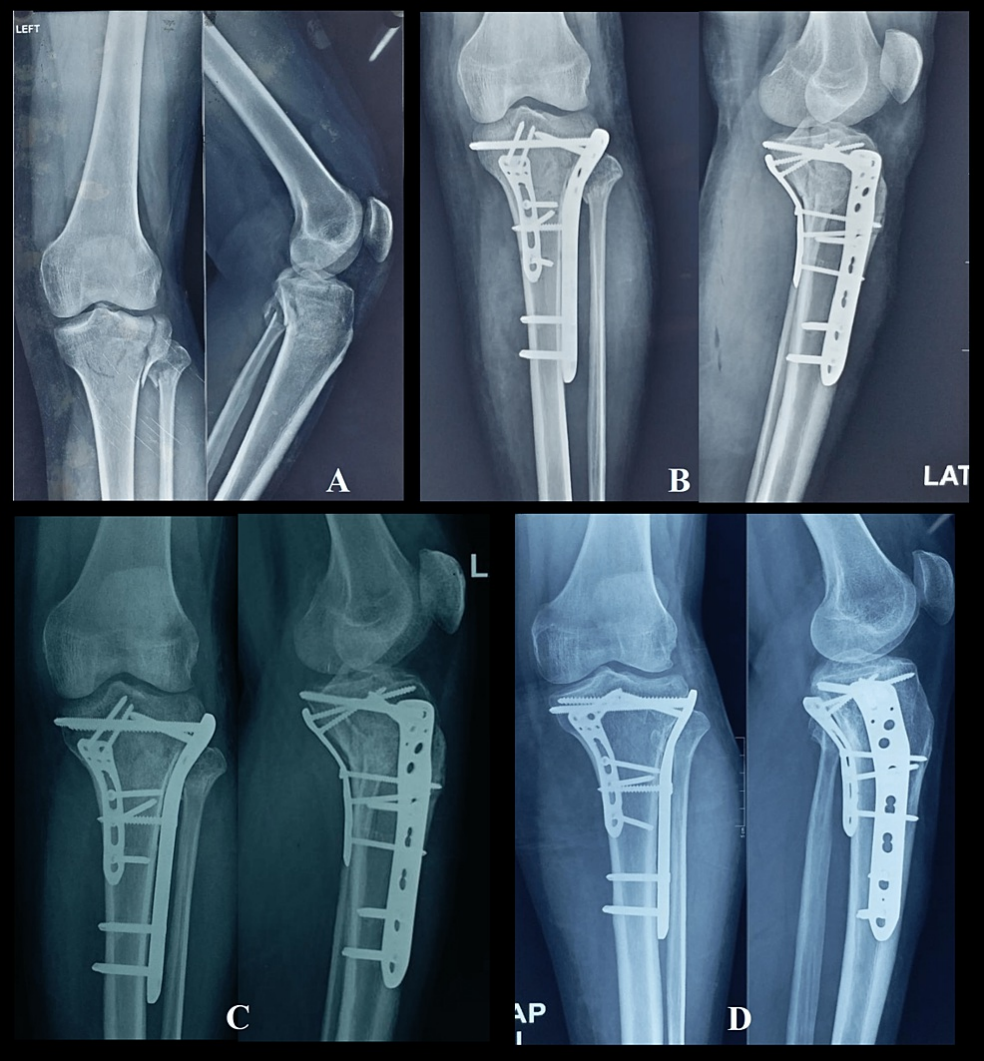
Serial follow-up radiographs of a 28-year-old male with complex proximal tibial plateau fracture of the left side managed by dual plating using two separate incisions. A: Pre-operative radiographs showing the sustained fracture pattern. B: Immediate postoperative radiograph of the fracture managed by anterolateral plate along with posteromedial buttress plating. C: Follow-up radiograph six months after surgery. D: Follow-up radiograph 12 months after surgery.

The serial radiographs of a case of a 28-year-old male who sustained a complex proximal tibial plateau fracture of the left side, which was managed by dual plating, are demonstrated in Figure 3.

**Figure 4:**
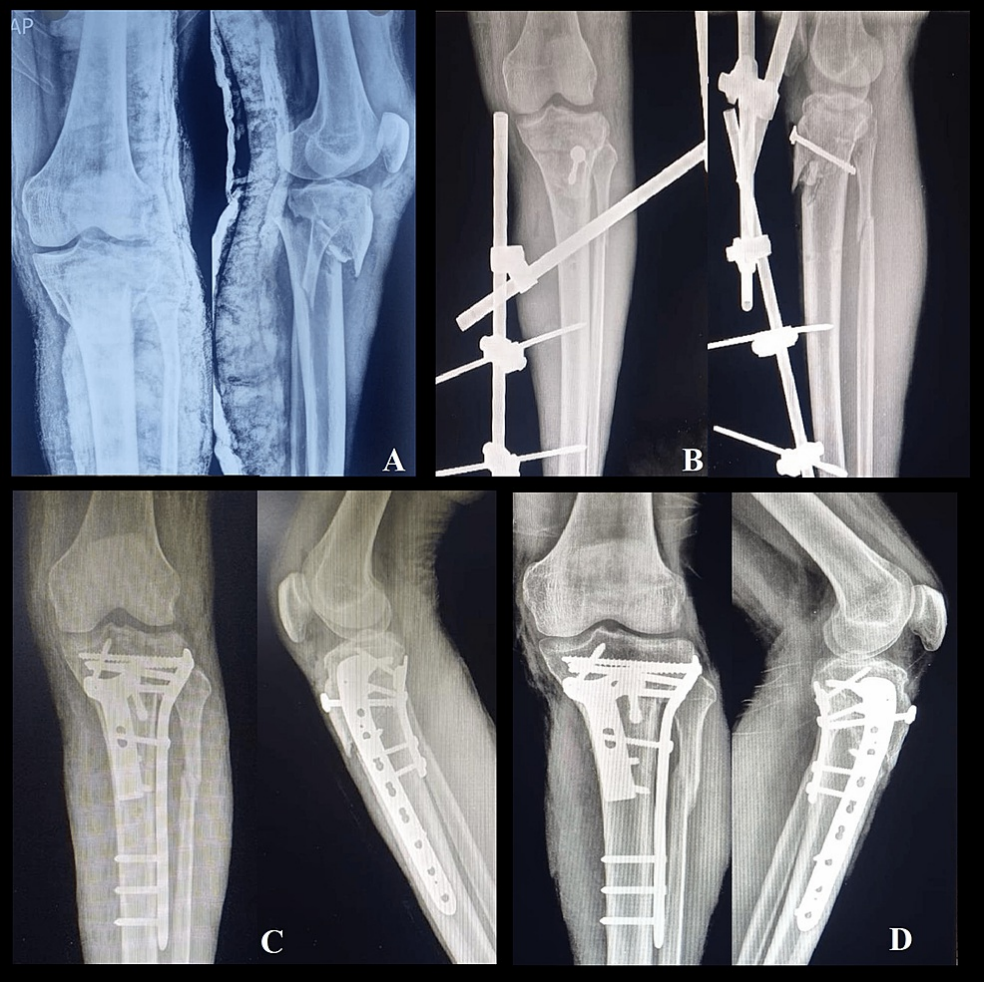
Serial follow-up radiographs of left side complex tibial plateau fracture in a 35-year-old male managed with staged procedures due to soft tissue compromise at presentation. A: Preoperative radiographs of the sustained fracture managed with primary splintage using a plaster-of-Paris slab. B: Postoperative radiographs after primary management with a temporary external fixator (associated fracture of the tibial tuberosity was primarily fixed with a corticocancellous screw). C: Radiographs at three-month follow-up after definitive management with dual plating using two different approaches after the subsidence of soft tissue swelling. D: Radiographs at the final follow-up 12 months postoperatively.

The sequential follow-up radiographs of a 35-year-old male with a complex proximal tibial plateau fracture of the left side managed by dual plating are depicted in Figure 4.

## Discussion

The management of CTPFs with PTPFs is challenging, considering soft-tissue complications, fracture comminution and morphology, and delayed complications such as varus collapse, stiffness, and arthritis [13-14]. Posterior plating for CTPFs having PTPF is becoming more popular, as research has shown that supplemental fixation of the posterior column with a posteromedial buttress plate results in the strongest fixation in terms of fracture stabilization [12, 15, 21]. The aim of the operative management of these fractures includes anatomic reduction of fracture fragments, maintenance of articular congruity, preservation of surrounding soft tissues, and avoidance of complications, particularly infection and malalignment. We aimed to employ the same principles in our study and found that this technique was easy, not very demanding, allowed appropriate visualization of fracture segments, and provided good to excellent functional outcomes in most cases.

Fixation with a single lateral locking plate fails in the following conditions: medial intraarticular fracture line, small comminuted medial plateau fragment, and medial articular fracture having a coronal component with a posteromedial fragment [3-5]. Higgins TF et al. reported that the incidence rate of posteromedial fragments in 111 patients with bicondylar tibial plateau fractures was 59% [5]. A single fixed-angle locking plate applied laterally may not effectively engage the posteromedial fragment making stable fixation of complex plateau fractures difficult. Schatzker type V and VI fractures require reduction and stabilization of both medial and lateral condyles of the tibia. The dual plate technique fixes both medial and lateral columns and restores mechanical stability with adequate fixation [11, 19-22]. It has been observed that the conventional anterolateral plate fixation with a supplementary posterior plate had superior biomechanical strength and less subsidence rate compared to a single lateral locking plate [21, 22]. The results reported by Jiang R et al. indicate a higher incidence of postoperative malalignment of the proximal tibia in fractures fixed with lateral less invasive stabilization system group than those in the dual plate group [23]. These results correlate with the present study, and no cases of collapse were observed during follow-up. Wang SQ et al. [14] also supported the finding that satisfactory results can be obtained in the treatment of CPTF when a posterolateral/posteromedial approach is used to fix the primary fragment in the posterior tibial plateau, followed by an anterior approach if knee stability is necessary (based on intraoperative assessment of the knee after posterior columnar fixation). Kim CW et al. [24] retrospectively reviewed the outcomes of 138 tibial plateau fractures managed with open reduction and internal fixation with plates and concluded that tibial plateau fractures with posterior coronal fracture components pose a challenge in their adequate rigid fixation with a single medial or lateral locked plate. Thereby leading to loss of fracture reduction and recommended either direct fixation of the fragments or buttressing them with a plate through the posteromedial, posterolateral, or posterior approach. Row ER et al. [25] performed a retrospective multicentric study including 28 knees with multicolumnar tibial plateau fractures having posterior fragments managed by a staged Lobenhoffer approach in the prone position, followed by supine anterolateral column access and reported excellent postoperative radiographic results and acceptable clinical outcomes. These findings are in accordance with the present study, which also showed good to excellent functional outcomes as measured by RFS in 24 (85.71%) patients and excellent OKS in 24 (85.71%) cases. One patient (3.57%) in our study was reported to have a secondary loss of reduction postoperatively at the final follow-up with an articular depression of more than 2 mm. However, the patient still achieved a good functional outcome without any further deterioration. This finding is comparable to the one observed by Söylemez MS et al., who also reported postoperative malreduction due to 2 mm articular depression in a single case (4%) out of a total of 25 patients [4-5].

The drawback of fixation with dual plating is the extensive soft tissue dissection which may increase the risk of wound complications. Various reports have reported complications such as infection, wound dehiscence, delayed wound healing, and skin necrosis [5,21,26,27]. Neogi DS et al. prospectively followed 32 patients managed with dual plating and reported superficial infection in four (12.5%) and deep infection in one (4%) patient [21]. Söylemez MS et al. observed a case of superficial infection in the anterolateral incision (4%), which healed with oral antibiotic treatment [4]. These findings do not vary significantly from our study, in which no cases of deep infection were seen. In contrast, superficial infection was reported in two patients (7.14%) who required oral antibiotics and recovered completely. These findings also correlate with other similar research conducted in India in which Sinha S et al. observed two cases of deep wound infection and one case of postoperative wound dehiscence among the 30 patients managed with dual plating [27]. In contrast, Prasad GT et al. observed one case with delayed wound healing and one with skin necrosis out of the total of 40 operated patients [26]. As infection is a common complication associated with dual plating, it is thus advised to handle the soft tissue gently and wait for 5 to 6 days after injury for tissue edema to subside and skin condition to improve. Another recent study by Raj M et al. reaffirms the findings that dual locking provides rigid fixation in bicondylar tibial plateau fractures with good functional and radiological outcomes without increasing the incidence of complications significantly [28]. To date, there is no consensus on the choice of an appropriate implant for fixing posterior column fractures in the literature. For PTPF fixation, some authors have used anatomical 3.5 mm T buttress plates, while others used semi-tubular plates or both [22,29,30]. In our study, both 3.5 mm T buttress plates and posteromedial locking plates have been used for PTPF fixation, and no cases of implant failure were seen.

Limitations

The greatest limitation of this study is that it is not a randomized trial with a control group and is a single-center study with a small number of cases due to the complex fracture pattern. There was no comparison with the target population, so there is little confidence in this technique being superior. A randomized controlled trial with a larger sample is needed to confirm the effectiveness of supplementary posterior plating using direct PM or PL incision. Furthermore, it may be difficult to operate in a prone position if the patient has concomitant chest, abdominal and pelvic injuries. Therefore, it is imperative to keep the risk versus benefit ratio in mind while utilizing this approach.

## Conclusions

Although treating CTPFs associated with PTPFs remains challenging even for skilled surgeons, the additional posteromedial plate to the conventional anterolateral plate for the stability of the posterior fragment prevents articular collapse. It achieves rigid fixation, a high knee score, a good range of motion, lower complication rates, and limited deformities with a good radiological outcome by the time of fracture union. The approach for posteromedial plating is technically demanding and incommodious due to changing the patient's position from prone to supine during surgery and are associated with minor complications like prolonged operative time, increased blood loss, and protracted hospital stay.

## References

[1] Mills WJ, Nork SE (2002). Open reduction and internal fixation of high-energy tibial plateau fractures. Orthop Clin North Am.

[2] Bhattacharyya T, McCarty LP 3rd, Harris MB, Morrison SM, Wixted JJ, Vrahas MS, Smith RM (2005). The posterior shearing tibial plateau fracture: treatment and results via a posterior approach. J Orthop Trauma.

[3] Mthethwa J, Chikate A (2018). A review of the management of tibial plateau fractures. Musculoskelet Surg.

[4] Söylemez MS, Cepni SK, Kemah B, Batar S (2022). Posteromedial plate application using medial midline incision for complex tibia plateau fractures: a retrospective study. BMC Musculoskelet Disord.

[5] Higgins TF, Kemper D, Klatt J (2009). Incidence and morphology of the posteromedial fragment in bicondylar tibial plateau fractures. J Orthop Trauma.

[6] Schatzker J, McBroom R, Bruce D (1979). The tibial plateau fracture. The Toronto experience 1968--1975. Clin Orthop Relat Res.

[7] Meinberg EG, Agel J, Roberts CS, Karam MD, Kellam JF (2018). Fracture and dislocation classification compendium-2018. J Orthop Trauma.

[8] Xiang G, Zhi-Jun P, Qiang Z, Hang L (2013). Morphological characteristics of posterolateral articular fragments in tibial plateau fractures. Orthopedics.

[9] Yoon RS, Liporace FA, Egol KA (2015). Definitive fixation of tibial plateau fractures. Orthop Clin North Am.

[10] Haase LR, Haase DR, Moon TJ (2022). Is pin-plate overlap in tibial plateau fractures associated with increased infection rates?. Injury.

[11] Lin KC, Tarng YW, Lin GY, Yang SW, Hsu CJ, Renn JH (2015). Prone and direct posterior approach for management of posterior column tibial plateau fractures. Orthop Traumatol Surg Res.

[12] Galla M, Lobenhoffer P (2003). [The direct, dorsal approach to the treatment of unstable tibial posteromedial fracture-dislocations]. Unfallchirurg.

[13] Carlson DA (2005). Posterior bicondylar tibial plateau fractures. J Orthop Trauma.

[14] Wang SQ, Gao YS, Wang JQ, Zhang CQ, Mei J, Rao ZT (2011). Surgical approach for high-energy posterior tibial plateau fractures. Indian J Orthop.

[15] Ruffolo MR, Gettys FK, Montijo HE, Seymour RB, Karunakar MA (2015). Complications of high-energy bicondylar tibial plateau fractures treated with dual plating through 2 incisions. J Orthop Trauma.

[16] (2006). Knee function-rasmussen. J Orthop Trauma.

[17] Dawson J, Fitzpatrick R, Murray D, Carr A (1998). Questionnaire on the perceptions of patients about total knee replacement. J Bone Joint Surg Br.

[18] Oztürkmen Y, Caniklioğlu M, Karamehmetoğlu M, Sükür E (2010). Calcium phosphate cement augmentation in the treatment of depressed tibial plateau fractures with open reduction and internal fixation. Acta Orthop Traumatol Turc.

[19] Hashemi J, Chandrashekar N, Gill B (2008). The geometry of the tibial plateau and its influence on the biomechanics of the tibiofemoral joint. J Bone Joint Surg Am.

[20] Pornrattanamaneewong C, Narkbunnam R, Chareancholvanich K (2012). Medial proximal tibial angle after medial opening wedge HTO: a retrospective diagnostic test study. Indian J Orthop.

[21] Neogi DS, Trikha V, Mishra KK, Bandekar SM, Yadav CS (2015). Comparative study of single lateral locked plating versus double plating in type C bicondylar tibial plateau fractures. Indian J Orthop.

[22] Zhang Y, Fan DG, Ma BA, Sun SG (2012). Treatment of complicated tibial plateau fractures with dual plating via a 2-incision technique. Orthopedics.

[23] Jiang R, Luo CF, Wang MC, Yang TY, Zeng BF (2008). A comparative study of Less Invasive Stabilization System (LISS) fixation and two-incision double plating for the treatment of bicondylar tibial plateau fractures. Knee.

[24] Kim CW, Lee CR, An KC, Gwak HC, Kim JH, Wang L, Yoon DG (2016). Predictors of reduction loss in tibial plateau fracture surgery: focusing on posterior coronal fractures. Injury.

[25] Row ER, Komatsu DE, Watson JT, Jones C, Kottmeier S (2018). Staged prone/supine fixation of high-energy multicolumnar tibial plateau fractures: a multicenter analysis. J Orthop Trauma.

[26] Prasad GT, Kumar TS, Kumar RK, Murthy GK, Sundaram N (2013). Functional outcome of Schatzker type V and VI tibial plateau fractures treated with dual plates. Indian J Orthop.

[27] Sinha S, Singh M, Saraf SK, Rastogi A, Rai AK, Singh TB (2019). Fixation of posterior tibial plateau fracture with additional posterior plating improves early rehabilitation and patient satisfaction. Indian J Orthop.

[28] Raj M, Gill S, Rajput A, Singh KS, Verma KS (2021). Outcome analysis of dual plating in management of unstable bicondylar tibial plateau fracture - a prospective study. Malays Orthop J.

[29] Eggli S, Hartel MJ, Kohl S, Haupt U, Exadaktylos AK, Röder C (2008). Unstable bicondylar tibial plateau fractures: a clinical investigation. J Orthop Trauma.

[30] Yoo BJ, Beingessner DM, Barei DP (2010). Stabilization of the posteromedial fragment in bicondylar tibial plateau fractures: a mechanical comparison of locking and nonlocking single and dual plating methods. J Trauma.

